# Low concentrations of serum testosterone predict acute myocardial infarction in men with type 2 diabetes mellitus

**DOI:** 10.1186/s12902-015-0034-1

**Published:** 2015-07-25

**Authors:** Bledar Daka, Robert D. Langer, Charlotte A. Larsson, Thord Rosén, Per Anders Jansson, Lennart Råstam, Ulf Lindblad

**Affiliations:** Department of Public Health and Community Medicine/Primary Health Care, University of Gothenburg, Gothenburg, Sweden; University of Nevada School of Medicine, Las Vegas, NV USA; Department of Clinical Sciences, Community Medicine, Lund, Sweden; Department of Endocrinology, Medicine, Göteborg, Sweden; Department of Internal Medicine, Medicine, Göteborg, Sweden

## Abstract

**Background:**

The aim of the present study was to investigate the associations between endogenous testosterone concentrations and the incidence of acute myocardial infarction (AMI) in men and women with and without type 2 diabetes.

**Methods:**

The study comprised 1109 subjects ≥40 years of age (mean age 62 ± 12 years) participating in a baseline survey in Sweden in 1993–94. Information about smoking habits and physical activity was obtained using validated questionnaires. Serum concentrations of testosterone and sex hormone-binding globulin (SHBG) were obtained using radioimmunoassay. Diagnosis of type 2 diabetes was based on WHO’s 1985 criteria. Individual patient information on incident AMI was ascertained by record linkage with national inpatient and mortality registers from baseline through 2011.

**Results:**

The prevalence of type 2 diabetes at baseline was 10.0 % in men and 7.5 % in women. During a mean follow-up of 14.1 years (±5.3), there were 74 events of AMI in men and 58 in women. In age-adjusted Cox models, a significant inverse association between concentrations of testosterone and AMI-morbidity was found in men with type 2 diabetes (HR = 0.86 CI (0.75–0.98)). In a final model also including waist-to-hip ratio, systolic blood pressure, total cholesterol and active smoking, the association still remained statistically significant (HR = 0.754 CI (0.61–0.92)).

**Conclusion:**

Low concentrations of testosterone predicted AMI in men with type 2 diabetes independent of other risk factors. Trials with testosterone investigating the effect regarding cardiovascular outcome are still lacking. Future trials in this field should take into account a modification effect of diabetes.

## Background

Serum concentrations of testosterone in men decrease with age [1]. Although the decrease may partially be attenuated by increasing levels of sex hormone binding globulin (SHBG) [2], obesity has a major independent impact on the decrease in these levels [3]. On the other hand, prospective studies have shown that low serum levels of testosterone can independently predict both obesity and diabetes [4]. This bidirectional relationship between low testosterone and metabolic syndrome as well as type 2 diabetes has been described in previous studies [5]. The link between concentrations of serum testosterone and type 2 diabetes has become a topic of debate as two prospective studies have shown that SHBG can also independently predict type 2 diabetes [6, 7].

Observational prospective studies and meta-analytical studies have also shown that low concentrations of testosterone predict both all cause and cardiovascular mortality in elderly populations [8–13]. There is an on-going debate regarding mechanisms behind this association, as it remains unclear whether concentrations of serum testosterone are a marker of aging in men, or whether testosterone per se influences the aging process and atherosclerosis [14]. In fact, studies conducted in men following an acute myocardial infarction (AMI) have demonstrated that testosterone induces vasodilatation, thus increasing coronary artery perfusion [15]. Moreover, accelerated atherosclerosis is observed in elderly castrated men [16]. However, epidemiological studies investigating the association between concentrations of serum testosterone and AMI are still scarce. This question may be especially important in individuals with type 2 diabetes who have accelerated atherosclerosis due to impaired glucose metabolism. Concentrations of testosterone are low in this group; however, it is not known whether low testosterone predicts greater risk of acute myocardial infarction. A recent trial investigating the effects of testosterone replacement therapy (TRT) in patients with type 2 diabetes has shown beneficial effects on insulin resistance (HOMA-ir) and on LDL-cholesterol [17]. Moreover, a previous meta-analysis has shown that TRT tends to improve glycaemic control and fat mass in individuals with type 2 diabetes [18]. Nonetheless, there is a lack of prospective studies investigating the association between concentrations of testosterone and AMI-morbidity in individuals with diabetes.

Apart from the general question of associations between concentrations of testosterone and cardiovascular diseases, knowledge is limited with regard to this association in women, and previous results are inconsistent [19–21]. A recent prospective study showed a strong association between free testosterone concentrations and cardiovascular disease (CVD) mortality in postmenopausal women with diabetes [22].

In light of these concerns, the present study aimed to investigate the associations between endogenous testosterone concentrations and the incidence of acute myocardial infarction in a population-based sample of men and women with and without type 2 diabetes.

## Research design and methods

### Study population

In brief, this study is based on a population-based random sample of men and women from Skara, a municipality in south-western Sweden. The study was conducted in 1993–94 and 1109 subjects were included if they completed a standard physical examination, filled in the study questionnaire, and provided venous blood for laboratory assessment. The overall participation rate was 80 %. Detailed information about this population sample is presented elsewhere [23].

### Medical history, socio-economic and life style factors

Standard questionnaires were used to collect information on previous hospitalizations and chronic diseases, and on smoking, alcohol habits, and leisure time physical activity (LTPA). Smoking was categorized as current, former or daily smoking.

### Physical examination

The physical examination included body height, weight, and waist and hip circumference. Waist-to-Hip Ratio (WHR) was calculated, as well as Body Mass Index (BMI). Nurses measured blood pressures after a 5 min rest by the study subjects, according to a standard protocol. Systolic and diastolic pressures were taken twice, one minute apart, with the subject in a supine position, at the right brachial artery and with the arm at heart level, using a cuff size appropriate for arm circumference and pressures recorded at the closest 2 mmHg [24].

### Clinical chemistry

Samples including plasma and serum were drawn after an overnight fast and were immediately frozen at − 82 °C. Fasting blood glucose and an oral glucose tolerance test in asymptomatic subjects with fasting blood glucose levels ≥6.7 mmol/L were the basis for diabetes mellitus diagnosis according to the WHO criteria [25]. Clinical criteria including age, body weight, symptoms at initial stage, tendency to ketoacidosis, and treatment were used to define type 1 and type 2 diabetes, respectively. Insulin resistance was estimated using the homeostatic model assessment for insulin resistance (HOMA-IR) [26]. Total cholesterol, HDL-cholesterol, LDL-cholesterol and serum-triglycerides were analysed using standard procedures.

Immunoassays were used to analyse concentrations of sex hormones. Total testosterone was analysed at Skåne University Hospital, Malmö, Sweden with Beckman Coulter kit: 2003, 386982A (CV = 7–8 %). Sex hormone-binding globulin (SHBG) (CV = 5 %) was analysed at Unilabs at Skaraborg Hospital, Skövde, Sweden, using Siemens Immulite kit 2000XPi [27]. Free testosterone and estradiol were calculated using albumin levels measured in the same venous sample, and SHBG, according to validated equations [28, 29]. The measurements of SHBG and testosterone were unsuccessful in 4 of 1109 subjects; those 4 were excluded from the analyses.

### Ethical considerations

All participants provided signed informed consent prior to enrolment, and the Regional Ethical Review Board in Gothenburg, Sweden, approved the study.

### Outcomes

All participants were followed from the baseline examination until a first cardiovascular event or death, or otherwise until December 31, 2011. All events were retrieved by record linkage with the Swedish Cause of Death and Hospital Discharge Registers, which is a valid alternative to revised hospital discharge and death certificates [30, 31] The outcomes considered in this study were non-fatal and fatal events of AMI (ICD8 and 9: 410; ICD10: I21) [32].

### Statistics

Analyses were performed using SPSS Statistics for Mac, version 20. Normal distribution of testosterone was observed, while Homa-ir and s-insulin had skewed distributions, and logarithmic variables were used when adjusting for these variables. Schoenfeld proportional hazards were used to determine the feasibility of Cox regression analysis. Cox Proportional Hazards Regression was employed to investigate the associations between levels of sex hormones at baseline and outcomes. Multivariate models were used to assess interactions and to estimate the roles of possible confounders. Stratified analyses for type 2 diabetes mellitus were computed to investigate possible effect modification by diabetes. All analyses were two-sided, and *p* < 0.05 was used as level of statistical significance.

## Results

The characteristics of the population at baseline are presented in Table 1. Men had significantly higher diastolic blood pressure and Homa-ir than women, while the prevalence of hypertension, type 2 diabetes, smoking, and AMI was non-significantly higher than in women at baseline. The differences in baseline characteristics between individuals with and without type 2 diabetes are presented in Table 2. Subjects with type 2 diabetes had higher systolic blood pressure, BMI, and WHR. At baseline, men with type 2 diabetes had lower testosterone concentrations than men without diabetes, but no differences in the levels of SHBG were observed. In both men and women, the baseline prevalence of hypertension and AMI was higher in participants with type 2 diabetes. However, there were fewer smokers among subjects with diabetes.

**Table 1:** Characteristics of the population at baseline

	Men		Women		
	Mean	SD	Mean	SD	p
Age (y)	62.2	12.4	61.8	12.4	0.898
BMI (kg/m2)	26.4	3.29	26.5	4.66	0.700
WHR	0.93	0.06	0.81	0.07	<0.001
FB-glc (mmol/L)	5.1	1.4	4.9	1.1	0.046
Serum insulin (μUI/ml)	6.64	5.99	6.46	9.17	0.554
Homa-IR	1.5	1.5	1.3	1.2	0.045
Systolic blood pressure (mmHg)	137	21.0	139	22.7	0.448
Diastolic blood pressure (mmHg)	78	9.9	75	11	<0.001
Cholesterol (mmol/L)	5.9	1.4	6.1	1.7	0.532
Hormones					
Testosterone (mmol/L)	13.5	4.36	1.00	0.62	<0.001
Free testosterone (mmol/L)	0.26	0.08	0.014	0.01	<0.001
SHBG (mmol/L)	39.2	16.5	54.4	26.1	<0.001
	N	%	N	%	
Hypertension	126	22	117	22	0.899
Type 2 Diabetes Mellitus	51	9.8	40	7.3	0.063
Smoking	115	23	101	19	0.071
History of AMI	37	6.9	27	4.7	0.137

General linear models were used to estimate the difference between men and women, respectively

*SD* standard deviation, *BMI* body mass index, *FB-glc* fasting blood glucose, *Homa-Ir* homeostasis model of assessment - insulin resistance, *BP* blood pressure, *AMI* acute myocardial infarction

**Table 2:** Differences at baseline between subjects with and without T2D, respectively

	Men			Women		
	No diabetes Mean (SD)	T2DM Mean (SD)	p	No diabetes Mean (SD)	T2DM Mean (SD)	p
Age y	62.2 (10)	70.5 (13)	<0.001	61.9 (10)	73.2 (13)	<0.001
BMI kg/m^2^	26.2 (3.2)	27.7 (4.4)	0.004	26.3 (4.5)	28.5 (5.1)	0.005
WHR	0.93 (0.1)	0.96 (0.1)	0.001	0.81 (0.1)	0.86 (0.1)	<0.001
FB-glc mmol/L	4.8 (0.6)	7.9 (3.8)	<0.001	4.8 (0.6)	7.7 (3.3)	<0.001
S-insulin μUI/ml	6.2 (4.6)	8.8 (12.6)	<0.001	5.7 (4.2)	9.2 (28.2)	<0.001
Homa-Ir	1.3 (1.2)	3.1 (2.8)	<0.001	1.2 (1)	3.2 (2.3)	<0.001
SBP mmHg	136 (17)	150 (18)	<0.001	138 (23)	152 (22)	<0.001
DBP mmHg	78 (10)	81 (9)	0.076	75 (11)	76 (11)	0.445
Cholesterol mmol/L	6 (1.1)	5.9 (1.3)	0.702	6.1 (1.3)	6.1 (1.3)	0.896
Hormones						
S-Testosterone mmol/L	13.7 (4.4)	11.7 (4.4)	0.002	1.0 (0.6)	1.2 (0.7)	0.047
S-SHBG mmol/L	39.7 (16)	40.2 (18)	0.845	54.9 (25)	47.7 (31)	0.077
Free testosterone mmol/L	0.26 (0.08)	0.21 (0.08)	<0.001	0.01 (0.01)	0.02 (0.01)	0.001
	N (%)	N (%)		N (%)	N (%)	
Hypertension	83 (17)	34 (63)	<0.001	105 (20)	21 (49)	<0.001
Smoking	113 (24)	5 (10)	0.021	101 (19)	2 (5)	0.017
History of AMI.	31 (6)	6 (11)	0.198	21 (4)	6 (14)	0.012

General linear models were used to estimate the difference between subjects with and without diabetes, respectively

*T2DM* type 2 diabetes, *BMI* body mass index, *FB-glc* fasting blood glucose, *Homa-Ir* homeostasis model of assessment - insulin resistance, *SBP* systolic blood pressure, *DBP* diastolic blood pressure, *AMI* acute myocardial infarction

**Fig. 1:**
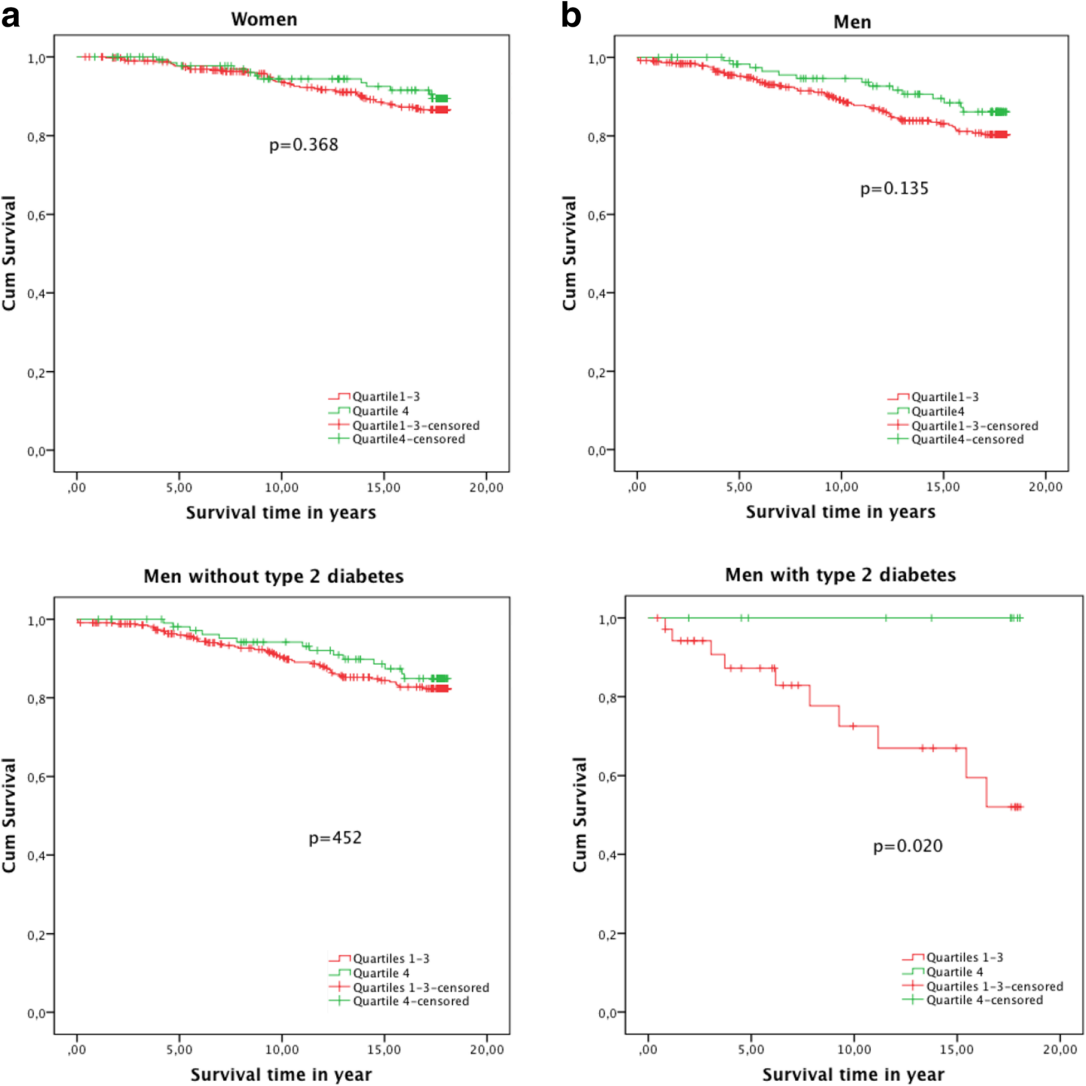
Kaplan-Meyers curves show the differences in the survival between subjects in the highest quartile in green line and the other three quartiles in red

The mean follow-up time was 14.1 ± 5.3 years. During that period, 74 AMI events occurred in men and 58 in women in the total sample. The event rate was 10.3 per 1000 person years in men and 6.9 per 1000 person years in women among participants without type 2 diabetes. Among the total AMI events, 10 AMIs occurred in men and 9 occurred in women, respectively, with diabetes. The event rate was 13.8 per 1000 person years in men with type 2 diabetes and 13.3 in women with type 2 diabetes. Kaplan-Meyer analyses for the highest quartile of testosterone versus the three lower quartiles stratified by gender were performed to investigate the association with incident AMI. In men, the Kaplan-Meyer curves were separated during the entire follow-up interval (Fig. 1), and stratified analyses were then conducted for men with and without type 2 diabetes. Among men with diabetes, the rate of AMI was significantly lower in the highest quartile of testosterone compared with the three lowest quartiles. Although the tendency was similar in men without diabetes, the findings in that group were not statistically significant.

Table 3 shows the results from the Cox models of the predictive effect of testosterone on AMI risk with adjustments for important covariates. In the model including all men regardless of diabetes status, the association between testosterone and AMI was marginally significant in the age-adjusted analysis (HR = 0.950 95%CI 0.90–1.00, *p* = 0.050). In the model adjusting for age, WHR, smoking habits, physical activity, LDL and systolic blood pressure, the association was not significant (HR = 0.949 95%CI 0.90–1.00 *p* = 0.069). An interaction term assessing the relationship between testosterone and type 2 diabetes was marginally significant (*p* = 0.051). When men with type 2 diabetes were analysed separately, a strong association was found in the age-adjusted model, which was further strengthened in the full model (HR = 0.754 95 % CI 0.61–0.92 *p* = 0.006). Models using free testosterone showed similar results (Table 3).

**Table 3:** Cox regressions analyses to investigate the association between testosterone and AMI

	Men			Women		
	HR	CI	p	HR	CI	P
T-T	All subjects					
	Adjusted for age					
	0.950	0.90–1.00	0.050	0.720	0.45–1.12	0.142
	Adjusted for age, WHR, smoking, physical activities, LDL, SBP					
	0.949	0.90–1.00	0.069	0.696	0.45–1.08	0.104
	Subjects with type 2 diabetes					
	Adjusted for age					
	0.828	0.71–0.97	0.022	0.759	0.24–2.39	0.638
	Adjusted for age, WHR, smoking, physical activities, LDL, SBP					
	0.754	0.61–0.92	0.006	0.647	0.18–2.28	0.498
F-T	All subjects					
	Adjusted for age					
	0.976	0.95–1.01	0.136	0.765	0.56–1.05	0.098
	Adjusted for age, WHR, smoking, physical activities, LDL, SBP					
	0.974	0.94–1.01	0.153	0.722	0.52–1.00	0.046
	Subjects with type 2 diabetes					
	Adjusted for age					
	0,886	0.81–0.97	0.012	0.581	0.27–1.25	0.163
	Adjusted for age, WHR, smoking, physical activities, LDL, SBP					
	0.856	0.77–0.96	0.006	0.579	0.26–1.31	0.190

HR = hazard ratio by 1 mmol/L-change in total testosterone concentration and by 0.01 mmol/L change in free testosterone. CI = 95 % of confidence interval

*T-T* total testosterone, *F-T* free testosterone, *WHR* waist hip ration, *LDL* low density lipoprotein, *SBP* Systolic blood pressure, *SHBG* sex hormone-binding globulin

In women, trends were similar with even lower point estimates (suggesting protection) for the associations between testosterone and AMI; however, the results were generally not statistically significant. One model assessing the relationship between free testosterone and AMI in all women with full covariate adjustment was marginally significant (HR = 0.722. 95 % CI 0.52–1.00, *p* = 0.046). Models testing the associations between SHBG and AMI showed no significant associations.

## Discussion

In this study, a strong and independent association between concentrations of testosterone and AMI was observed in men with type 2 diabetes. A trend was also observed in the entire cohort regarding an inverse association between serum testosterone and AMI. The association in men was stronger than the association in women, and it remained significant after adjustment for age. The association was strongest among men with type 2 diabetes, where it remained significant with adjustment for age and factors within the metabolic syndrome.

Previous studies in elderly men have shown an association between low levels of testosterone and higher risk for CVD [8, 10, 13]. While the evidence in elderly men is very robust [11–13], prospective studies in younger men are lacking. The cardiovascular impact of low testosterone in this group might be difficult to detect because of the low event rate. Another explanation might be a modifying effect of age with regard to the testosterone effects in blood vessels. The levels of testosterone might be more important in blood vessels where ageing has occurred. This hypothesis is supported by our findings. In fact, in the current data, the major impact of testosterone was observed among subjects with type 2 diabetes where early vascular ageing occurs [33]. However, repeated measurements of testosterone concentration would provide more information regarding the effects of testosterone during ageing. Low testosterone concentrations have previously been associated with the metabolic syndrome [4]. There is evidence supporting an inverse relationship between testosterone levels and the risk of developing type 2 diabetes and obesity [4].

However, the inverse relationship between testosterone and incident AMI in men with type 2 diabetes remained significant after the adjustment for age and metabolic risk factors, suggesting other mechanisms to be involved. One possibility is an effect on the coronary perfusion through actions on the artery wall. Fukui et al. have reported that low concentrations of endogenous androgens were associated with increased artery stiffness in men with type 2 diabetes [29, 34]. A similar result was reported by Akishita et al. [35] in a study of the association between endothelial function and testosterone levels. Moreover, Web et al. [15] have shown a vasoactive effect of testosterone in men with coronary disease, supporting the idea that testosterone might have an independent vasoprotective effect. In contrast with these findings, a recent observational study in the US showed that hypogonadal men on testosterone replacement therapy had a higher incidence of cardiovascular events than controls [36]. However, the retrospective design of the study could not address possible selection bias, as individuals with a higher cardiovascular risk might have been more likely to be treated with testosterone.

The present study showed that in women, free testosterone had a greater impact on the risk of AMI than endogenous testosterone. This may be an artefact of the laboratory methods used, as the assays for testosterone were developed to assess testosterone in men and optimised for the male range, and thus less accurate in women and children [37]. Furthermore, testosterone production declines with the slowing of ovarian function in menopause; however, this was not possible to take into consideration here, as information about menopause at baseline was lacking. It should also be mentioned that this was a post-hoc analysis. Trials investigating the effects of testosterone on the risk for cardiovascular disease are lacking.

Another limitation of our study is the small number of men with type 2 diabetes, as there is a risk of overestimating effects in subgroup analyses [38]. However, the consistency of these results across strata and the significant findings despite the small sample size warrant further investigation. The population-based sample and high participation rate are major strengths of the study. The long follow-up is also a strength, and the divergence of the survival curves (Fig. 1) during the follow-up period suggests a consistent relationship. Another strength is the availability of a wide variety of risk factors at baseline, which permitted adjustment for established associations and assessment of possible confounding.

## Conclusions

In conclusion, measuring testosterone in men with type 2 diabetes may help in the assessment of their cardiovascular risk. Larger studies estimating the effects of low testosterone levels in men with diabetes in relation to AMI are needed. This study evaluated endogenous testosterone, and whether exogenous testosterone can reduce AMI risk in men with diabetes and low concentrations of testosterone also remains to be studied.
